# Research Progress of Natural Small-Molecule Compounds Related to Tumor Differentiation

**DOI:** 10.3390/molecules27072128

**Published:** 2022-03-25

**Authors:** Xiaoli He, Yongkang Liao, Jing Liu, Shuming Sun

**Affiliations:** 1 Molecular Biology Research Center, School of Life Sciences, Central South University, Changsha 410078, China; hexiaoli@csu.edu.cn (X.H.); liaoyongkang@csu.edu.cn (Y.L.); 2 Hunan Province Key Laboratory of Basic and Applied Hematology, School of Life Sciences, Central South University, Changsha 410078, China

**Keywords:** tumor, differentiation, leukemia, solid tumor, natural small-molecule compounds

## Abstract

Tumor differentiation is a therapeutic strategy aimed at reactivating the endogenous differentiation program of cancer cells and inducing cancer cells to mature and differentiate into other types of cells. It has been found that a variety of natural small-molecule drugs can induce tumor cell differentiation both in vitro and in vivo. Relevant molecules involved in the differentiation process may be potential therapeutic targets for tumor cells. Compared with synthetic drugs, natural small-molecule antitumor compounds have the characteristics of wide sources, structural diversity and low toxicity. In addition, natural drugs with structural modification and transformation have relatively concentrated targets and enhanced efficacy. Therefore, using natural small-molecule compounds to induce malignant cell differentiation represents a more targeted and potential low-toxicity means of tumor treatment. In this review, we focus on natural small-molecule compounds that induce differentiation of myeloid leukemia cells, osteoblasts and other malignant cells into functional cells by regulating signaling pathways and the expression of specific genes. We provide a reference for the subsequent development of natural small molecules for antitumor applications and promote the development of differentiation therapy.

## 1. Introduction

Cancer is one of the major diseases threatening human health in the 21st century. Statistical studies have shown that 19.3 million new cancer patients were diagnosed, and 10 million died from cancer in 2020. If the national incidence rate stays the same, there will be 28.4 million cancer patients worldwide in 2040—47% higher than in 2020 [1]. A tumor is a new organism formed by local tissue and cell proliferation under the action of various tumorigenic factors. Because the new organism mostly presents space-occupying massive processes, tumors are also known as vegetation. In addition to uncontrolled cell proliferation and survival, the continuous loss of normal differentiation is also a feature of most malignant cells [2]. Malignant tumors include carcinoma, sarcoma, hematopoietic and lymphoid malignancies, etc. The conventional treatments of tumors include surgery, radiotherapy, chemotherapy, targeted therapy, immunotherapy, traditional Chinese medicine therapy, etc., which are aimed at killing tumor cells. However, killing tumor cells inevitably damages normal cells, and tumor cells cannot be eliminated due to their migratory and invasive nature. Tumor differentiation is a method aimed at reactivating the endogenous differentiation program of cancer cells and inducing cancer cells to stop proliferation and restore normal cell characteristics [3]. The molecular mechanisms involved in the differentiation process are potential therapeutic targets for tumor cells, and the induction of differentiation is an ideal outcome for both chemopreventive and therapeutic agents.

Based on the fact that a tumor is a disease with disturbed cell differentiation and impaired maturation, attempts to induce cancer cells to redifferentiate towards maturity into structurally and functionally normal cells have gradually started to move from the laboratory to the clinic. In 1961, Pierce first found that mouse testicular teratoma cells could spontaneously differentiate into benign normal cells, which verified that malignant tumors could be cured by inducing tumor cell differentiation, as proposed in the 1950s, and research was initiated to induce tumor cell differentiation [4]. Then in 1975, Friend reported that dimethyl sulfoxide induced erythrocyte differentiation and stimulated hemoglobin synthesis in mouse leukemia cells, which confirmed the possibility of inducing differentiation in vitro [5]. In addition, Wang Zhengyi found that symptoms of acute promyelocytic leukemia (APL) were completely relieved by all-trans retinoic acid (ATRA) in 72% of patients in 1985, creating a precedent for the clinical application of differentiation-inducing agents in the treatment of leukemia [6]. Now, APL can be cured by the combined treatment of retinoic acid (RA) and arsenic.

According to the mechanisms of tumor differentiation, differentiation inducers can be divided into endogenous and exogenous agents. The former refers to the chemicals with the differentiation-inducing effect produced by tumor or host cells, such as colony-stimulating factors and glucocorticoids; and the latter refers to the differentiation inducers that cannot be produced by tumor or host cells but must rely on external supply, including vitamins, nucleosides and their analogs, organic compounds, etc. At present, a variety of compounds inducing the differentiation of cancer cells have been reported, such as theophylline [7], trimeric glutaryl pentane [8] and 5,7-dimethoxycoumarin [9], which can induce the differentiation of blood tumor cells or solid tumor cells by arresting their proliferation through diverse mechanisms. More research shows that a large number of differentiation-inducing agents have been found and synthesized all over the world.

However, the above agents also have certain limitations, such as causing adverse reactions and drug resistance. It has been found that natural small-molecule compounds widely present in plants, such as polyphenols, flavonoids, carotenoids, alkaloids, etc., have a broad safety profile and therapeutic effects on a wide range of diseases; therefore, some of them have entered clinical research, and many strategies have been proposed for their improvement [2,10,11]. Myeloid leukemia cells are particularly sensitive to most of these natural small-molecule compounds, which are induced to differentiate into morphologically and functionally mature cells. The use of natural small-molecule compounds to induce malignant cell differentiation represents a more targeted and potentially less toxic treatment of oncological disease, which could provide hope for improved treatment of malignancies by avoiding the significant side effects seen with conventional therapies.

**Figure 1:**
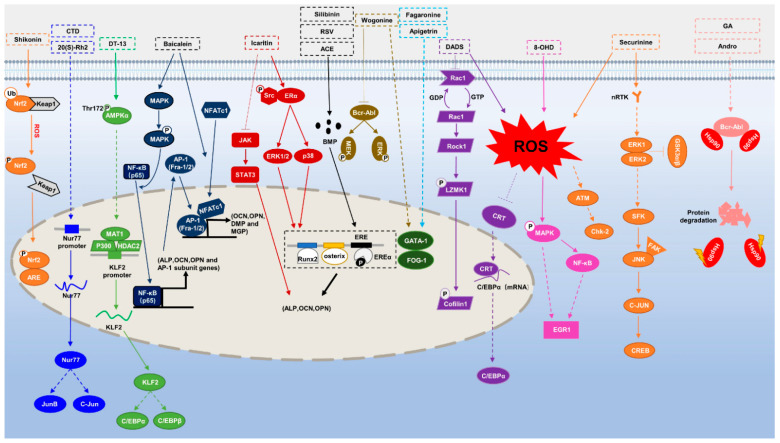
Mechanism of differentiation-inducing action of natural antitumor compounds.

This review focuses on natural small-molecule compounds that induce myeloid leukemia cells, osteoblasts, and some other solid tumor cells to advanced stages of differentiation by regulating signaling pathways and the expression of specific genes. In particular, we aim to summarize the current status of research on natural small-molecule compounds that show differentiation induction of malignant cells and their mechanisms (several natural compounds with a detailed mechanism of action are shown in Figure 1) and to provide basic knowledge to facilitate the development of differentiation therapies. We used “small molecules”, “natural compounds”, “tumors”, “hematologic tumors” or “solid tumors” and “differentiation” or “differentiation therapy” or other relevant items as keywords in a search of the PubMed database. In addition, the Clinicaltrials.gov database was searched for relevant diseases to obtain clinical trials of natural compounds. No restrictions were set on the date of publication of the literature.

## 2. Hematologic Tumors

Hematologic tumor is a general term for a large group of malignant tumors that originate in the hematopoietic system. Common hematological tumors include various types of leukemia, multiple myeloma and malignant lymphoma. Hematological malignancies are a group of heterogeneous hematological tumors usually characterized by abnormal production of blood cells (hematopoiesis) [12]. Although the clinical management of hematologic malignancies has improved significantly over the past few years, the socioeconomic costs are a concern. Meanwhile, many key challenges remain, such as recurrence, refractory lesions, and morbidity and mortality associated with malignancy. If tumor cells can be induced to differentiate and prevented from proliferating, their malignant potential could be controlled. Therefore, differentiation therapy is a promising treatment method. A successful example of this method is all-trans retinoic acid (ATRA) against acute myeloid leukemia, which promotes the implementation of differentiation therapy. It is worth noting that many natural small molecule compounds have shown significantly therapeutic potential in promoting differentiation in myeloid leukemia (Table 1).

### 2.1. Myeloid Leukemia

Leukemia is a kind of malignant clonal disease of hematopoietic stem cells. Its incidence rate is high (about 27,600 per 100,000 people) because of its diverse pathogenic factors [13]. According to the data of global cancer statistics in 2020, leukemia ranks 15th among all types of cancer, with 474,519 new cases (2020) and 311,594 deaths [1]. The classification of leukemias is more complex, and it is often clinically classified into myeloid and lymphocytic leukemias based on different series of leukodystrophies. Myeloid leukemias can in turn be divided into acute myeloid leukemia (AML) and chronic myeloid leukemia (CML) based on the rapidity of the condition and the maturation of leukemic differentiation, of which AML is the most common, accounting for more than 50%.

#### 2.1.1. Acute Myeloid Leukemia (AML)

Acute myeloid leukemia (AML) is mainly caused by chromosome translocation, which produces fusion proteins with abnormal activity, resulting in cell-cycle imbalance and failure of hematopoietic differentiation. The consequence of the replacement of normal hematopoietic stem cells with tumor cells is a decrease in the number of blood cells, anemia, thrombocytopenia and neutropenia. Although current treatment strategies provide a reasonable possibility for the majority of patients to achieve complete remission, the recurrence rate and subsequent disease-related mortality remain high [2]. In 2020, there were 19,940 newly diagnosed AML cases, and 11,180 patients died of AML in the United States [14]. The improvement of chemotherapy and maintenance therapy increased the overall survival rate of young patients (less than 15 years old) to more than 60%; however, in the end, 40% of people relapse and need salvage therapy.

Induction of cell differentiation has also become an important choice in cancer therapy due to the cyclic imbalance and abnormal hematopoietic differentiation characteristics of AML. The potential of AML differentiation agents has also been demonstrated through the use of all-trans retinoic acid (ATRA), which has achieved significant clinical success in a small number of AML patients with the acute promyelocytic leukemia (APL) subtype [15,16]. In addition to synthetic drugs, a variety of plant-derived compounds (phytochemicals) induce cell differentiation through a variety of mechanisms and show antitumor activity. Many natural small molecules contribute to mediation of apoptosis, cell cycle and differentiation through oxidative stress pathways. Reactive oxygen species (ROS) are one of the key markers of differentiation; in this case, they are considered potential therapeutic agents for the treatment of AML. Securinine is an alkaloid from the root of the plant *securinega suffruticosa*. Securinine activates receptor tyrosine kinase (nRTK) to induce HL-60 differentiation towards monocytes, with increased expression of CD11b and CD14. Not only does securinine decrease the expression of transcription factors, such as CCAAT/enhancer-binding proteins α (C/EBPα), C/EBPε, GATA-1 and cellular-myelocytomatosis viral oncogene (c-myc), but it also initiates ROS-induced DNA damage by activating the JNK/ERK pathway, which induces ATM/ATR and Chk1-dependent cell differentiation. More importantly, securinine has also been reported to induce AML tumor differentiation in primary leukemia patients, as well as in nude mice, showing potent differentiation activity and potential for clinical application. At present, securinine has been used for clinical treatment of neurological disorders, although it occasionally induces epilepsy due to GABA. GABA activation does not adversely affect securinine’s activity to induce AML differentiation. Therefore, this alkaloid could be considered as a potential therapeutic agent of AML [17,18,19,20]. Coincidentally, diallyl disulfide (DADS), a major anticancer active ingredient derived from garlic, can also induce leukemia cell differentiation via ROS pathways in which CRT was downregulated and translocated, resulting in the release of the creatine transporter (CRT) and C/EBP α mRNA interactions that promote C/EBP α protein expression. It was previously believed that DADS exerted anticancer activity by defending against carcinogen invasion and inhibiting proliferation of various cancer cells, but the latest study showed that, apart from ROS pathways, DADS could also negatively regulate the rac1-rock1-limk1-cofilin1 axis to induce leukemia cell differentiation to play an anticancer role. In addition, DADS significantly inhibited the growth and induced the differentiation of HL-60 cells in SCID mice [21,22,23,24]. Small-molecule natural compounds that induce cancer cell differentiation via ROS pathways also include shikonin and ethyl acetate extract (CAE). Shikonin, a major component of the herb comfrey, may interact with biological targets through the formation of covalent bonds or as an electron transfer agent in redox reactions to regulate redox homeostasis between cells via the Nrf2/ARE pathway, thereby promoting differentiation and apoptosis in HL-60 cells. Low doses (<100 ng/mL) of paclitaxel shift cells from proliferation to differentiation; however, higher concentrations lead to cell death [25,26,27]. Another small-molecule natural compound is ethyl acetate extract (CAE), an ethyl acetate extract from *Caesalpinia sappan L*, which differentially inhibits AML cells in vitro in a concentration-dependent manner without toxic effects on normal cells and induces mitochondrial apoptosis, G2/M-phase cell cycle arrest and late cell differentiation through reactive oxygen species [28].

Studies have shown that orphan nuclear receptor Nur77 is an important tumor suppressor gene in AML and that deletion of Nur77 expression is a common feature in patients with AML. Deletion of Nur77 leads to impaired differentiation of hematopoietic stem cells and bone marrow progenitor cells, which contributes to development of AML. Targeted activation of Nur77 expression has been shown to be a potential new intervention approach in the treatment of AML. Ginsenoside 20(s)-Rh2 is a protopanaxadiol-type ginsenoside and also one of the typical components of red ginseng. Ginsenoside 20(s)-Rh2 [29,30,31] promotes Nur77 translocation from the nucleus to the mitochondria by activating the Nur77-mediated death receptor pathway (Fas and DR5), followed by Nur77 interaction with Bcl-2 to cause apoptosis. 20(S)-Rh2 induces AML cell differentiation via the Nur77-mediated transcription factors c-Jun and JunB [32,33]. Like 20(s)-Rh2, CTD also induces apoptosis and differentiation through a Nur77-mediated signaling pathway. Cantharidin (CTD) is the main bioactive component of cantharidin. Recent studies have found that CTD and its derivatives, such as Cantharidic acid and norcantharidin, inhibit leukemia cell proliferation, induce apoptosis, cause cell cycle arrest and enhance the inhibitory effect of chemotherapeutic drugs [34,35,36]. Notopterol, a coumarin, is an active monomer extracted from *N. incisum* with antipyretic, analgesic, and anti-inflammatory properties. Notopterol [37,38,39] induces apoptosis, differentiation and G0/G1 cell cycle arrest in human AML HL-60 cells. Notopterol markedly induces protein expression of c-Jun and JunB and decreases c-myc, thereby inducing differentiation [40]. DT-13 is a new compound isolated from *Liriope muscari* (Decne) Baily, which has strong cytotoxicity to a variety of solid tumors. Similar to 20(S)-Rh2, Liriodendron chinense saponin C (DT-13) [41,42,43] induces apoptosis in HL-60 and Kasumi-1 cells by upregulating Fas, FasL, DR5 and TRAIL through the expression of Kruppel-like factor 2 (KLF2). The restoration of KLF2 by DT-13 is highly correlated with AMPK-related histone acetylation mechanisms. DT-13 significantly enriches Ace-H3 (AH3-120) in the promoter region of the KLF2 gene. In addition, KLF2 promotes differentiation by upregulating the expression levels of the differentiation markers CD11b and CD14 and the transcription factors C/EBPα and C/EBPβ. Therefore DT-13 may mediate apoptosis and differentiation through AMPL-KLF2 [44]. DT-13 is classified by the Chinese Ministry of Health as an herbal medicine due to its high efficiency and safety.

#### 2.1.2. Chronic Myeloid Leukemia (CML)

Chronic myeloid leukemia (CML) is characterized by the abnormal accumulation of immature leukemia mother cells in blood, bone marrow and spleen, which prevents the terminal differentiation of myeloid cells but promotes the expression of BCR-ABL fusion oncoprotein. BCR-ABL fusion protein plays a role in the imbalance of tyrosine kinase activity, which activates proliferation and antiapoptotic signal pathways and leads to the malignant expansion of pluripotent stem cells in bone marrow. Furthermore, BCR-ABL fusion protein mediates downregulation of C/EBPα expression, which triggers a block in the myeloid differentiation of CML cells. By inducing terminal differentiation, leukemic cells may lose their ability to proliferate and form mature, functional cells. Therefore, induction of differentiation is an ideal therapeutic strategy.

Unlike AML, CML cells are particularly sensitive to erythrocyte differentiation. Many natural compounds, including apigenin and fagaronine or other anthracycline antibiotics and HSP90 inhibitors, have been proved to induce CML cell differentiation into erythroid lineage in vitro. These inducers work through different mechanisms, including direct or indirect inhibition of BCR-ABL. Almost all these compounds activate the key erythroblast transcription factor GATA-1. Apigenin [45,46] is considered to be a bioactive flavonoid with anti-inflammatory and antioxidant effects. It upregulates the mRNA expression of the erythroid transcription factor GATA-1 and alters the nuclear and cytoplasmic DTP distribution, thereby affecting the nuclear translocation and localization of signaling molecules, such as GATA-1. Long-term treatment with apigenin induced erythroid-like differentiation of K562 cells. Analysis of the structure–function relationships of the flavonoids showed that apigenin-induced differentiation of K562 cells was due to the 2–3 double bonds and hydroxyl groups in their structure. Apigenin mainly acts as a glucoside conjugate due to its unstable pure form, and its conjugated form may be an important determinant of its bioavailability and absorption [47,48]. Fagaronine, a benzo phenanthrene alkaloid extracted from *Fagara zanthoxyloides* Lam, exerts its differentiation activity by specifically activating genes involved in the regulation of GATA-1 in the region of erythroid phenotypic expression [49]. Wogonine [50,51,52] is a flavonoid extracted from *Scutellaria baicalensis* Georgi that has a good curative effect in the treatment of hematological malignancies. Wogonine inhibited BCR-ABL-mediated phosphorylation of MEK and ERK in K562 cells and increased the expression of erythroblast differentiation genes by upregulating transcription factor GATA-1 and enhancing the combination between GATA-1 and friend of GATA-1 (FOG-1). Differentiation was accompanied by cell cycle arrest in the G0/G1 phase, as well as concomitant upregulation of p21 and downregulation of CDK4 and cyclin D1 [53].

Small-molecule tyrosine kinase inhibitors (TKIs) [54], such as imatinib, dasatinib, nilotinib and ponatinib, have been developed to treat CML by blocking the kinase structural domain of the BCR-ABL oncoprotein. However, there are a variety of adverse reactions of these compounds, such as off-target and metabolic toxicity, due to the resistance of BCR-ABL mutation to TKI treatment and the progression to advanced diseases. It is necessary to seek new treatment strategies. Gambogic acid (GA) is a small molecule derived from the Chinese herb Garcinia cambogia. It promotes apoptosis and differentiation in imatinib-resistant chronic granulocytic leukemia cells through induction of proteasome inhibition and caspase-dependent BCR-ABL downregulation. Studies on GA derivatives found that GA derivatives lacking reactive α, β-unsaturated carbonyl groups were ineffective, suggesting that α, β-unsaturated carbonyl groups are important for the exertion of GA activity [55,56,57]. Andrographolide (Andro), a major component of the medicinal plant *Andrographis paniculata*, promotes the differentiation of imatinib-sensitive and imatinib-resistant CML cells through the production of ROS, which prompts BCR-ABL downregulation and Hsp90 cleavage. The aliphatic ester enhances the cytotoxic activity of Andro and the double bond between C-8 and C-17, in addition to the lactone and its conjugated double bond, maybe the central structure in which it acts [58]. In general, Andro and GA provide an alternative and complementary strategy for imatinib-resistant CML by inhibiting BCR-ABL function through a different mechanism than that of imatinib [59,60,61]. 8-hydroxydaidzein (8-OHD, 7,8,4′-trihydroxyisoflavone) is a hydroxylated derivative of daidzein isolated from fermented soybean products. The o-dihydroxy group in the 8-OHD structure has superior antioxidant and free radical scavenging activity and may be the key moiety for its action. 8-OHD activates the mitogen-activated protein kinase (MAPK) and nuclear clearance factor-κB (NF-κB) signaling pathways, thereby inducing autophagy, apoptosis and cycle arrest. In addition, 8-OHD induces degradation of BCR-ABL oncoprotein and promotes early growth response 1 (EGR1)-mediated megakaryocyte differentiation, which could be a potential compound for the treatment of CML [62,63]. Atractylodes macrocephala (also known as white bamboo) is an important component of several traditional Chinese medicine prescriptions used to treat abdominal pain and gastrointestinal diseases for thousands of years. The main active compound, atractylenolide I (ATL-I), induces apoptosis and differentiation of human leukemia cells [64].

**Table 1:** Myeloid leukemia differentiation therapy induced by natural compounds.

Tumor	Compound	Source	Target	Structure	IC_50_/GI_50_/Concentration of Induction of Differentiation	Clinical Trial (Phase)	Reference
AML	Securinine	*Securinega suffruticosal*	ATM/ATK, chk1	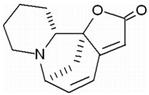	15 μM (HL-60 cells)	/	[17,18,19,20]
	DADS	Garlic	ROS, rac1-rock1-limk1-cofilin 1	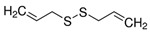	1.25 mg/L (HL-60 cells)	/	[21,22,23,24]
	Shikonin	Arnebia	Nrf2/ARE	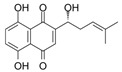	100 ng/mL (HL-60 cells)	Hepatitis C (Phase 2), HCV Recurrence After Liver Transplantation (Phase 2)	[25,26,27]
	CAE	*Caesalpinia sappan* L.	ROS	/	0.19 mg/mL (HL-60 cells), 0.15 mg/mL (Kasumi-1 cells)	/	[28]
	20 (s)-Rh2	Ginseng	Nur77	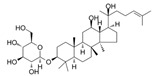	25.59 μM (HL-60 cells) and 60.06 μM (Kasumi-1 cells)	/	[32,33]
	CTD	Cantharides	Nur77	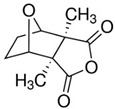	6.21 μM (HL-60 cells) and 8.00 μM (Kasumi-1 cells)	Molluscum Contagiosum Skin Infection (Phase 4)	[34]
	Notopterol	*N. incisum*	c-Jun, JunB	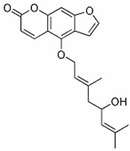	40.32 μM (HL-60 cells), 56.68 μM (Kasumi-1 cells) and 50.69 μM (U937 cells)	/	[40]
	DT-13	*Liriope muscari* (Decne.) Baily	AMPKα	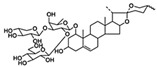	17.04 μM (HL-60 cells) and 19.34 μM (Kasumi-1 cells)	/	[32,44]
CML	Apigetrin	Celery	GATA-1	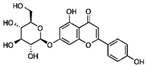	50 μM (K562 cells)	/	[47,48]
	Fagaronine	*Fagara zanthoxyloides* Lam	GATA-1	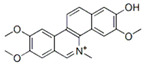	>10 μM (K562 cells)	/	[49]
	Wogonine	*Scutellaria baicalensis*	GATA-1, FOG-1	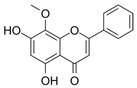	80 μM (K562 cells)	/	[53]
	GA	gamboges	Bcr-Abl	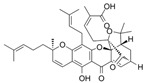	0.24 µmol/L (KBM5 cells), 0.34 µmol/L(KBM5-T315Icells) and 0.62 µmol/L (K562clls)	/	[55,56,57]
	Andro	*Andrographis paniculata*	Bcr-Abl	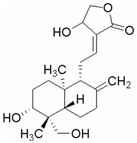	23.66 μM (K562 cells), 8.50 μM (KBM5 cells) and 8.50 μM (KBM5R cells)	Acute Tonsillitis (Phase 4), Acute Bronchitis (Phase 4)	[59,60,61]
	8-OHD	Soybean	MAPK, NF-κB	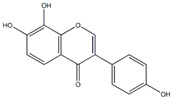	91.8 μM (24 h) and 49.4 μM (48 h) (K562 cells)	/	[62,63]
	ATL-I	*Atractylodes macrocephula*	CD14, CD68	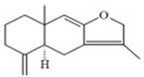	44.67 μg/mL (24 h), 37.63 μg/mL (48 h), 24.44 μg/mL(72 h) Jurkat cell and 4.23 μg/mL (24 h, 48 h, 72 h) (U937) cells	/	[64]

## 3. Multiple Myeloma (MM) and Osteosarcoma

Multiple myeloma (MM) is a malignant tumor in which plasma cells proliferate abnormally. MM can cause organ damage, including osteolytic lesions, anemia, renal failure or hypercalcemia (clonal expansion of malignant plasma cells). To date, multiple myeloma is still considered incurable, accounting for about 10% of all hematological malignancies and 2% of all cancers (Cancer Research UK, 2018). In 2016, there were 138,509 incident cases worldwide (Cowan, 2018). Between 1990 and 2016, the incidence rate of the whole world increased by 126% and was closely related to age (Cancer Research UK 2018; Cowan 2018) [65].

In MM disease, clonal malignant plasma cells accumulate in bone marrow, reduce osteoblast formation and stimulate osteoclasts to destroy the bone. Osteoblasts build bones by forming connective cell populations, and osteoclasts are large multinucleated cells that destroy bones. The functional balance of the two cell types is essential for bone maintenance and repair. Therefore, in addition to targeting myeloma cells to treat MM patients, osteoclasts and osteoblasts are also regarded as potential targets. At present, it has been proposed that drugs that induce osteoblast differentiation can be used as an alternative therapy for MM treatment. Osteoblast differentiation requires the activity of transcription factor 2 (Runx2) and Osterix transcription factors, as well as growth factors.

In MM therapy, natural small-molecule compounds act as differentiation agents for osteoblasts (Table 2). This study shows that resveratrol, silibinin, acerogenin A and baicalein can regulate the expression and activity of specific markers, including bone morphogenetic proteins (BMPs) and key transcription factors Osterix and Runx2, to induce differentiation. Bone morphogenetic proteins (BMPs), members of the TGF-β superfamily, are often the key regulators of bone formation and remodeling, and BMPs also play an important role in the differentiation of hBMSCs into osteoblast-like cells [66]. Silibinin is a strong antioxidant with a very strong hydrogen bonding of the 5-OH group to the adjacent oxo group, which is conjugated to the aromatic ring and acts as a free electron pair donor for the hydrogen bonding to the 5-OH group. Silibinin enhances osteoblast differentiation of human bone marrow stromal cells by inducing the expression of BMPs and activating BMP and Runx2 pathways through hydrogen bonding [67,68]. Acerogenin A (ACE) is a natural compound isolated from *Acer nikoense* Maxim. In MC3T3-E1 and RD-C6 cells and primary osteoblasts, ACE stimulates osteoblast differentiation via BMP action, which is mediated by Runx2-dependent and Runx2-independent pathways. In addition, ACE increases the expression of osteocalcin mRNA in which the hydroxyl groups of C-9 and C-11 may play a role [69,70]. Baicalein is a coumarin-like derivative extracted from Chinese herbs that induces early osteoblast differentiation through activation of the MAP kinase/NF-κB signaling pathway, and this activation is associated with increased expression of osteoblast differentiation markers. In addition, baicalin induced the differentiation of MC3T3-E1 in mouse osteoblasts [71,72,73,74,75]. Resveratrol (RSV) is a natural compound present in various plant species that reduces the growth of myeloma cell lines (RPMI 8226 and OPM-2) in a dose-dependent manner and inhibits ligand-receptor activator (RANKL)-induced osteoclast differentiation. In addition, RSV induced the expression of the osteoblast markers osteocalcin and osteoprotegerin in immortalized human bone marrow mesenchymal stem cells (HMSC-TERT), leading to osteoblast differentiation [76,77,78,79]. Several studies on RSV and its natural or synthetic analogues have highlighted the importance of 3,4-dihydroxyl groups in the expression of cytotoxic and proapoptotic activity, suggesting that hydroxyl groups are important for the expression of RSV activity. The RSV derivative STR50 is currently being tested in phase II clinical trials for the treatment of MM. Icaritin is the main constituent of *Herba Epimedii*. It has obvious inhibitory effects on a variety of blood cancer cells, including acute myeloid leukemia (AML), chronic myeloid leukemia (CML), multiple myeloma (MM) and lymphoma. By inhibiting the JAK-STAT pathway, icaritin induces cell differentiation, inhibits tumor cell migration and inhibits the growth of cancer stem/progenitor cells. Besides, icaritin has an estrogen-like chemical structure, which can induce MC3T3-E1 cell differentiation through estrogen receptor-mediated activation of ERK1/2 and p38 signaling [80,81,82,83,84]. Similarly, osteoblast differentiation and mineralization induced by the estrogen receptor (ER) pathway can also be used to treat osteosarcoma. The ER functions primarily as a DNA-binding transcription factor that regulates gene expression.

Osteosarcoma is the most common non-hematological skeletal malignancy in both children and adults [85]. Although modern treatment schemes have combined multiple approaches, such as chemotherapy, surgery and radiotherapy, the five-year survival rate of osteosarcoma patients has been maintained at 60–70% since the 1970s [86,87]. Osteosarcoma has a high rate of metastasis and chemoresistance and therefore has a poor prognosis. Although surgical techniques and implants have shown a steady progress, current chemotherapeutic drugs associated with toxic side effects, including cardiotoxicity, infertility and renal insufficiency, seem to be similar to those used 40 years ago [88]. This highlights the urgent need for new therapies and drugs. Osteosarcoma cells share many similar characteristics with undifferentiated osteogenic progenitor cells, including high proliferative capacity, resistance to apoptosis and similar expression profiles of many osteogenic markers, such as connective tissue growth factor, Runx2, alkaline phosphatase (ALP) and Osterix. Because more than 80% of osteosarcomas have histopathological differentiation differences, new therapies based on non-cytotoxic induction of cell differentiation response pathways may have good prospects.

Flavonoids are mainly derived from vegetables and herbs, which are widely found in nature and have a very rich pharmacological activity. Recent studies have shown that both natural and synthetic flavonoids show significant antitumor activity and are chemically similar to estrogens, and some have been used as alternatives to estrogens [89]. In addition to playing an important role in the reproductive system, estrogens also play an important role in bone metabolism. The osteoprotective effects of estrogens are mainly attributed to their inhibition of bone resorption and stimulation of bone formation. Quercetin is one of the main flavonoids. Some recent studies have shown that quercetin promotes osteoblast differentiation [90,91,92,93]. Moreover, several flavonoids, including icaritin [94,95,96,97,98], genistein [99,100,101,102] and kaempferol [103,104,105], have been shown to promote osteogenic differentiation by regulating the expression of Runx2 and BMP-2 differentiation through their estrogenic effects. For example, ugonin K is a flavonoid isolated from *helminthochys zeylanica* (L.) that induces osteoblast differentiation through activation of the non-classical SRC signaling pathway and the estrogen receptor-dependent classical pathway. It also efficiently induces cell differentiation and mineralization in MC3T3-E1 mouse osteoblasts [106,107]. In addition, TGF-β1 has targeted induction of osteogenic differentiation, which may represent a novel treatment strategy for osteosarcoma with fewer side effects. Galangin, a natural flavonoid product, inhibits human osteosarcoma cell growth by inducing transforming growth factor-β1-dependent osteogenic differentiation [108,109,110]. Similarly, the flavonoid hyperoxides inhibit the proliferation of osteosarcoma cells by inducing G0/G1, and the TGF-β signal pathway stimulates its osteogenic differentiation [111,112,113]. Coleusin factor is a diterpenoid isolated from the roots of the tropical plant *Coleus forskohlii*. It can inhibit the growth of osteosarcoma by inducing bone morphogenetic protein-2 (BMP-2)-dependent differentiation [114]. The hormonally active form of vitamin D, 1,25-dihydroxyvitamin D3 (VD3), also has potent antiproliferative, proapoptotic and prodifferentiation effects in many malignancies. VD3 and its analogs, such as osteosarcoma cell lines MG-63 and SaOS2, also promote osteoblast differentiation. P73 is essential for VD3-mediated osteosarcoma differentiation, and induction of p73 in response to DNA damage enhances VD3-mediated osteosarcoma cell line differentiation [115,116,117,118,119,120].

**Table 2:** Multiple myeloma (MM) and osteosarcoma differentiation therapy induced by natural compounds.

Tumor	Compound	Source	Target	Structure	IC_50_/GI_50_/Concentration of Induction of Differentiation	Clinical Trial (Phase)	Reference
MM	Silibinin	silymarin	BMP, Runx2	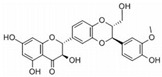	20 μmol/L (hBMSCs)	Transplantation (Phase 2), Hepatitis C (Phase 2)	[67,68]
	ACE	*Acer nikoense* Maxim	BMP	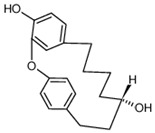	30 μM (MC3T3-E1 cells)	/	[69,70]
	Baicalein	*Scutellaria baicalensis*	NF-κB	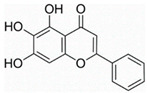	30 μM (U266 cells)	Influenz (Phase 2)	[71,72,73,74,75]
	RSV	Peanuts, grapes (red wine), tiger nuts, mulberries	RANKL	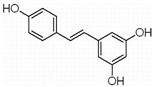	100 μmol/L(RPMI 8226, OPM-2 cells)	Multiple Myeloma (Phase 2), Liver Cancer (Phase 2)	[76,77,78,79]
	Icaritin	*Herba epimedii*	ERK1/2, p38	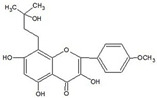	10 μM (MC3T3-E1 subclone14 cell)	Hepatocellular Carcinoma (Phase 2), Metastatic Breast Cancer (Phase 1)	[80,81,82,83,84]
Osteosarcoma	Quercetin	Rutin (rutin), quercetin, hypericin and other plants	ERα, Runx2, Osterix	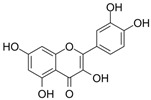	2.5 μM (BMSCs)	Diabetes (Phase 2), Alzheimer’s Disease (Phase 2)	[90,91,92,93]
	Icaritin	*Herba epimedii*	ERα, ALP, BMP2, Runx2, RANK-RANKL	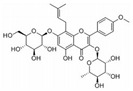	1 μM (primary osteoblasts)	Hepatocellular Carcinoma (Phase 2),- Metastatic Breast Cancer (Phase 1)	[94,95,96,97,98]
	Genistein	Legumes and bean products	BMP2/ SMAD5/Runx2	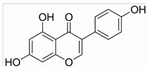	10^−6^ M (hBMSCs)	Sepsis (Phase 4), Metabolic Syndrome (Phase 3)	[99,100,101,102]
	KFL	*Kaempferia galanga* L.	ALP, ERα Runx2, Osterix	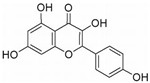	10 μM (Osteoblasts)	/	[103,104,105]
	Ugonin K	*Helminthostachys zeylanica* (L.)	SRC, ERα Runx2, Osterix	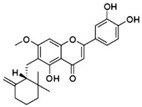	10 μM (MC3T3-E1)	/	[106,107]
	Galangin	*Alpinia officinarum*	TGF-β1/Smads	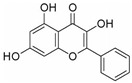	67.32 μM (MG-63 cells), 57.09 μM (U-2 OS cells)	/	[108,109,110]
	Hyperoside	hypericumperforatum	TGF-β, BMP2, OPN, Runx2	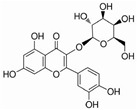	223.5 μM (U2OS cells), 239.0 μM (MG63 cells)	/	[111,112,113]
	Coleusin	Root of *Coleus forskohlii*	BMP-2	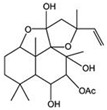	100 μmol/L (U2OS cells), MG63 cells)	/	[114]
	Vitamin D3	Animal offal, beef, lamb	p73	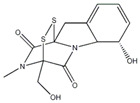	200 nmol/L (Osteoblasts)	Vitamin D Deficiency (Phase 4), Pancreatitis, Chronic (Phase 4)	[115,116,117,118,119]

## 4. Other Tumors

Many natural small-molecule compounds have antitumor and differentiation-promoting effects in a variety of tumors (Table 3). For example, kaempferol and genistein promote not only osteoblast differentiation but also melanoma differentiation. Melanoma is a type of cancer caused by the malignant transformation of melanocytes. Melanin production is thought to be a major marker of differentiation in melanoma models. For melanoma, many cell lines considered to represent the “blocked” stage of melanoma differentiation have been isolated and studied. Finding new cancer-specific differentiation inducers and clarifying the basis of cancer cell differentiation provides support for the development of improved melanoma therapy. An early study showed that theophylline (1,3-dimethylxanthine; Theo) induced the maturation and differentiation of B16 melanoma cells by activating cAMP-dependent protein kinase A and inducing phosphorylation of cAMP response element-binding proprotein (CREB) [121,122,123]. Isoliquiritigenin (ISL) from *licorice* can induce the differentiation of mouse melanoma B16F10 cells [3,124,125,126]. Recent studies have shown that three isopentenyl flavonoids in GF-1, GF-4 and GF-9 in licorice roots can significantly induce the differentiation of B16-F10 melanoma cells. In addition, kaempferol [127,128,129,130,131], genistein [132,133,134] and 3′3-diindolylmethane (DIM) [135,136] inhibit the viability of melanoma cells and induce apoptosis and differentiation of malignant melanoma cells mediated by reactive oxygen species and endoplasmic reticulum stress [137].

Neuroblastoma is the most common extracranial solid tumor in children, accounting for about 15% of all cancer-related deaths in children [138]. The prognosis is related to patient age, MYCN oncogene amplification and tumor differentiation. Retinoic acid (RA) is used as a differentiation agent for maintenance therapy of high-risk neuroblastoma (NB), but its use is limited by its related toxicity. Natural compounds can avoid the limitations of toxicity and have great potential. Kaempferol (KFL) induces differentiation of neuroblastoma cells via the IRE1α-XBP1 pathway [139,140]. Melatonin (N-acetyl-5-methoxytrimethylamine) is an endocrine hormone mainly released by the pineal gland. Melatonin promotes the differentiation of neuroblastoma cells by activating cell phagocytosis induced by hyaluronate synthase 3 (HAS3) [141,142,143,144,145]. *Tinospora cordifolia* (TCE) has a strong ability to resist metastasis and induce differentiation in neuroblasts, and IMR-32 cells can be induced by treatment with higher concentrations of TCE. Studies have shown that TCE or phytochemicals derived from this plant can be used as safe pharmacological agents together with conventional drugs [146,147]. Chlorogenic acid (CA) is a plant compound isolated from *Eucommia ulmoides*, *honeysuckle* and other plants. In glioma cells, CA increases the expression of specific differentiation biomarkers Tuj1 and GFAP to induce differentiation. The therapeutic effect of CA in glioma cells is equivalent to that of temozolomide [148,149,150].

Glioblastoma is the most common and invasive primary brain tumor. It is the most malignant brain tumor, with a very poor prognosis. The current standard of care for patients with GBM includes tumor resection, followed by temozolomide radiotherapy and chemotherapy. However, due to the highly invasive nature of the tumor, it is almost impossible to remove completely. In addition, stem-like tumor cells resistant to chemotherapy and radiotherapy were found to regroup in the tumor cavity, resulting in tumor recurrence and drug resistance. The prognosis of patients with GBM remains very poor, with a median survival of 9.9 to 15 months.

Differentiation therapy has been proposed as a promising strategy for GBM treatment because after differentiation, GBM cells lose tumorigenicity and become sensitive to chemotherapy and radiotherapy [151]. Resveratrol promotes differentiation and inhibits the tumorigenic and self-renewal capacity of glioma stem cells (GSCs). Studies have demonstrated that Nanog is a key factor in the maintenance of GSCs and that resveratrol induces phosphorylation-dependent activation of p53, leading to Nanog degradation in GSCs, which may overcome treatment resistance in GSCs [152,153,154,155]. Xanthone-rich extract from *Gentiana dinarica* transformed roots, and its active component, norswertianin, induce autophagy and ROS-dependent differentiation of the human glioblastoma cell line [156,157]. Similarly, curcumin [158,159] can significantly induce differentiation of glioma-initiating cells (GICs) in vivo and in vitro through the induction of autophagy. Thus, curcumin can be used as an adjuvant to conventional chemotherapy in the treatment of GBM [160]. Falcarindiol (FAD) is a polyacetylene found in many dietary plants and has shown a variety of physiological activities, such as anti-inflammatory, antibacterial and hepatotoxic inhibition. FAD has an anticancer effect on glioblastoma cells by inducing the differentiation of glioblastoma stem cells and activating the apoptosis pathway of glioblastoma cells [161].

**Table 3:** Other tumor differentiation therapy induced by natural compounds.

Tumor	Compound	Source	Target	Structure	IC_50_/GI_50_/Concentration of Induction of Differentiation	Clinical Trial (Phase)	Reference
Melanoma	Theo	*Camellia sinensis* (L.)	MEK1/2, Wnt/β- Catenin	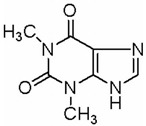	2 mM (B16 cells)	Asthma (Phase 3), Leukemia (Phase 2)	[121,122,123]
	ISL	Licorice	MAPK	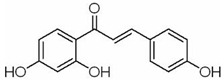	10 μM (A375, A2058 cells)	/	[3,124,125,126]
	KFL	*Kaempferia galanga* L.	m-TOR/PI3K/AKT, ERs, ROS	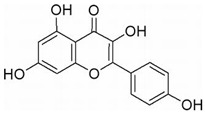	20 μM (A375 cells)	/	[127,128,129,130,131]
	Genistein	Soybean	FAK/paxillin, MAPK	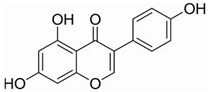	12.5 μM (B16F10)	Sepsis (Phase 4), Metabolic Syndrome (Phase 3)	[132,133,134]
	DIM	Cabbage, Brussels sprouts and cabbage	PTEN/Akt	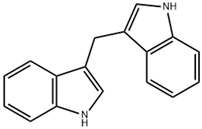	150 μM (A375 cells)	Breast Cancer (Phase 3)	[135,136]
Neuroblastoma	KFL	*Kaempferia galanga* L.	IRE1 α	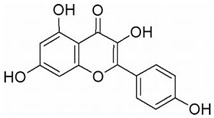	50 μM (IMR32 and Neuro2a cell)	/	[139,140]
	Melatonin	Pinecone	hyaluronic acid synthase 3	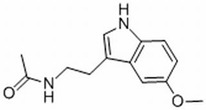	0.1 nmol/L (N2a cels)	Coronary Artery Calcification (Phase 4), cancer Malignancies (Phase 3)	[141,142,143,144,145]
	TCE	*Tinospora cordifolia*	NF200, MAP-2, NeuN	/	200 μg/mL(IMR-32 cells)	/	[146,147]
	CA	*Eucommia almoides oliver* or *Lonicera confuse*	Tuj1, GFAP	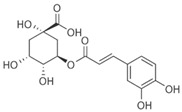	50 µM (Huh7 cells)	Type 2 Diabetes Nonalcoholic Fatty Liver (Phase 3), Advanced Lung Cancer (Phase 2)	[148,149,150]
Glioblastoma	RSV	Peanuts, grapes (red wine), tiger nuts, mulberries	p53/p21	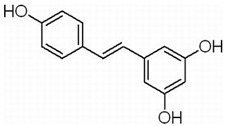	50 μM (GSCs)	Multiple Myeloma (Phase 2), Liver Cancer (Phase 2)	[152,153,154,155]
	Norswertianin	*Gentiana dinarica* transformed roots	Akt/mTOR, ROS	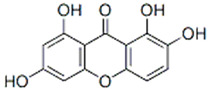	86.44 µM (U251 cells)	/	[156,157]
	Curcumin	*Curcuma longa*	autophagy	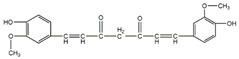	2 μM (SU-2, SU-3)	Type 2 Diabetes (Phase 4), Multiple Myeloma (Phase 2)	[158,159,160]
	FAD	North Sea Cucumber	Notch	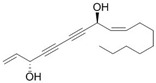	40 μM (U373 cells)	/	[161]

## 5. Discussion

One of the most important characteristics of cancer is the loss of differentiation; therefore, cancer is considered to be a disease of cell differentiation. Differentiation-related preclinical models were developed as early as the 1980s [162]. The concept of inducing cancer cell differentiation and cell proliferation arrest has become an alternative to cytotoxic chemotherapy. The purpose of this therapy is not to eliminate cells through cytotoxic and non-selective drug activity but to regulate the expression of signaling pathways and specific genes to guide cancer cells into higher stages of differentiation and reverse growth/differentiation, thereby reprogramming malignant cells into functional cells with different subtoxic doses.

**Figure 2:**
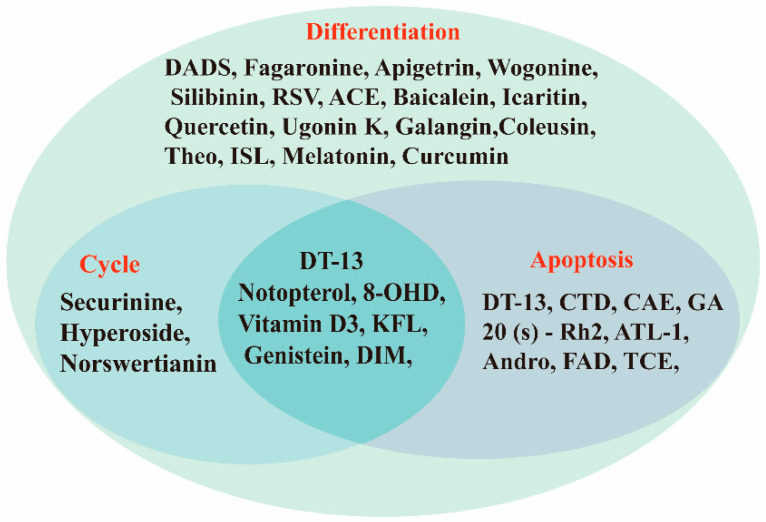
Natural small-molecule compound mechanisms of tumor inhibition.

In recent years, a large number of natural compounds have been reported, with good activity in tumor prevention and treatment, inducing tumor cell differentiation and apoptosis through a variety of mechanisms and inhibiting tumor cell cycle and proliferation (Figure 2). The mechanisms by which natural antitumor compounds work against tumors vary, but in general, they directly induce the expression of proteins associated with tumor cell differentiation or differentiation of cells with blocked differentiation, leading to antitumor effects. The natural small-molecule compounds used for the treatment of AML, such as securinine, CAE, DADS, etc., induce apoptosis and differentiation by inducing intracellular oxidative stress. In addition, 20(s)-Rh2, CTD, DT-13 and notopterol inhibit tumor cell proliferation and induce apoptosis and differentiation through cell cycle arrest. Compared to AML, natural small-molecule compounds exhibit different molecular mechanisms in CML cells, especially sensitivity to erythroid differentiation. Fagaronine, apigenin, and wogonin induce erythroid differentiation or indirectly inhibit BCL-ABL, activating the key erythroid factor GATA-1 and increasing the expression of erythroid differentiation genes. There are also many natural small-molecule compounds, such as GA, andro, 8-OHD and ATL-I, which play an antitumor role by inducing apoptosis and differentiation of CML cells. In MM disease, the accumulation of clonal malignant plasma cells in the bone marrow reduces osteoblast formation and stimulates osteoclasts to destroy bone. RSV, ACE, silibinin and baicalein have been shown to enhance osteoblast differentiation of human bone marrow stromal cells by regulating the expression and activity of specific markers, including bone morphogenetic proteins (BMPs) and other key transcription factors. Induction of osteoblast differentiation can therefore be used as an alternative therapy to MM treatment. Meanwhile, induction of osteoblast differentiation is also an effective treatment for osteosarcoma. In the treatment of osteosarcoma, several flavonoids, including ugonin K, icaritin, genistein and KFL, promote osteoblast differentiation through their estrogenic effects, whereas galangin and hyperoside induce osteogenic differentiation by targeting TGF-β1. Furthermore, hyperoside and vitamin D3 induce osteogenic differentiation by inhibiting the entire cell cycle process, inducing apoptosis in osteosarcoma cells and driving the cells to a more differentiated phenotype. A significant proportion of natural small-molecule compounds that induce differentiation of tumor cells are also found in other types of tumors. Studies have shown that KFL, genistein and DIM induce apoptosis and differentiation of malignant melanoma cells mediated by reactive oxygen species and endoplasmic reticulum stress through their inhibitory effects on melanoma cell viability. In addition, theophylline and ISL induce B16 melanoma cell maturation and differentiation. CA, KFL, melatonin and TCE promote neuroblastoma cell differentiation, and norswertianin, FAD and curcumin promote GBM cell differentiation. Among the 36 natural antitumor compounds mentioned in this article, 25 have in vivo anticancer effects, 15 are in clinical trials and one is FDA-approved for use.

In general, although many methods and techniques such as surgery, radiotherapy, chemotherapy, targeted therapy, immunotherapy and Chinese medicine therapy have been used for tumor treatment, most patients still face problems, such as disease recurrence and drug resistance. The development and research of natural small-molecule compounds provide a good solution for prolonging the remission period and improving the survival rate. Natural compounds are an important resource for modern drug development. From 1981 to 2019, 59% of newly approved small-molecule drugs (1123 in total) were derived from natural compounds and their derivatives [163,164]. Natural-molecule medicine and its preparations used under the guidance of modern medical theory have their unique advantages in treatment. Firstly, natural drugs come from nature, with wide sources and few toxic side effects. The material basis and in vivo mechanism of action of natural drugs are relatively clear, quality-controlled and safe, and natural drugs have shown high antitumor activity [165]. In addition, under the guidance of modern drug theory, natural drugs modified and transformed in structure have relatively concentrated targets, enhanced efficacy and richer forms of clinical application. For example, the combined use of modified and transformed natural molecule medicine with conventional anticancer drugs can provide relevant advantages of therapeutic efficacy by sensitizing malignant cells to drugs and overcoming drug-induced resistance in cancer.

Nevertheless, natural small molecules have several shortcomings. In many preclinical studies, pharmacokinetics and pharmacodynamics have not been well addressed, especially when natural compounds are combined with conventional drug therapy. This is because the heterogeneity and often combinatorial nature of natural compounds make it difficult to distinguish between the cause and the exact mechanism of action and exact information on the target in the numerous experimental studies, which once hampered research into the development of natural antitumor drugs. Most phytochemicals exhibit poor stability in their natural form. Some natural small-molecule compounds may also cause side effects, including headache, dizziness, nausea, diarrhea and liver dysfunction. The most important barrier to the clinical use of natural small molecules is that most natural small molecules are fat-soluble, and their low solubility in water and poor internal absorption and rapid metabolism contribute to their low bioavailability. Despite this, many scientists are still actively involved in the development and research of natural small-molecule drugs and have provided some current and future strategies for the development of effective and safe natural antitumor drugs.

1. Using medicinal chemistry, natural small-molecule compounds can be structurally modified and optimized to obtain new chemical entities with high drug resistance and low toxic side effects.
2. Using computer simulations of the drug screening process to predict the likely activity of compounds allows multiple biologically relevant pathways to be probed in a target-agnostic manner, which allows for targeted screening and greatly reduces the number of compounds to be experimentally screened, thereby shortening development cycles and saving money.
3. The development of innovative target-prediction tools to help identify biomolecular targets or potential off-target effects of drugs may help to determine the biological activity of natural products and guide the biochemical screening of natural products to reduce the number of experiments and save valuable resources.
4. Establishing sound testing methods and experimental approaches to promote drug development, improving methods for purification of active ingredients in natural compounds and extracting key components for subsequent experiments.
5. The search for or development of lead compounds for novel drugs with improved pharmacokinetic profiles through the study of natural product molecules with specific active backbones, active moieties and excellent biological activity.
6. Diverse and well-established animal or neural models are established for screening, development and validation.

Although much research has been conducted to treat cancer, the incidence and mortality of cancer continue to rise, and effective treatment remains a formidable challenge. The drugs currently used for treatment have very strong adverse side effects, such as vomiting, bone marrow suppression and liver dysfunction. Inducing the differentiation of malignant cells into functional cells is an effective alternative approach to treating tumors, where natural small-molecule compounds play an important role in inducing tumor cell differentiation due to their wide availability and low toxicity. However, problems encountered in clinical studies need to be addressed to improve the use of natural compounds in effective cancer treatment. Overall, natural small-molecule compounds, as new anticancer properties and mechanisms continue to be revealed, hold outstanding promise for future cancer differentiation therapy.

## 6. Conclusions

In this paper, we summarized various natural small-molecule compounds inducing differentiation in myeloid leukemia cells, osteoblasts and some solid tumor cells. This may promote the research of differentiation therapy, improve therapeutic efficiency and reduce drug resistance and cytotoxic side effects, etc.

## Abbreviations

ACE	Acerogenin A
ALP	Alkaline phosphatase
AML	Acute myeloid leukemia
Andro	Andrographolide
APL	Acute promyelocytic leukemia
ATL-I	Atractylodes lactone I
ATRA	All trans-retinoic acid
BC	Blast crisis
BMP-2	Bone morphogenetic protein-2
BMPs	Bone morphogenetic proteins
CA	Chlorogenic acid
CAE	Ethyl acetate extract
CAPE	Caffeic acid phenethyl ester
C/EBPα	CCAAT/Enhancer-Binding Proteins -α
CCND2	cell cycle protein D2
CDK6	Cyclin-dependent kinase 6
CML	Chronic myeloid leukemia
c-myc	Cellular-myelocytomatosis viral oncogene
CTD	Cantharidin
DADS	Diallyl disulfide
DIM	3′3-diindolylmethane
DT-13	Liriodendron chinense saponin C
EGR1	Early growth response 1
ER	Estrogen receptor
FAD	Falcarindiol
FDA	Food and Drug Administration
FOG-1	friend of GATA-1
GA	Gambogic acid
GATA-1	GATA Binding Protein 1
GBM	Glioblastoma
GICs	Glioma-initiating cells
HAS3	Hyaluronate synthase 3
ISL	Isoliquiritigenin
KFL	Kaempferol
KLF2	Kruppel-like factor 2
MAPK	Mitogen activated protein kinase
MM	Multiple myeloma
NB	Neuroblastoma
NF-κB	Nuclear factor-κB
OCN	Osteocalcin
RA	Retinoic acid
ROS	Reactive oxygen species
RSV	Resveratrol
RTK	Receptor tyrosine kinase
Runx2	Transcription factor 2
TBM	Tubeimosides
TCE	Tinospora cordifolia
TGF-β	transforming growth factor beta
Tho	Theophylline
TKIs	Tyrosine kinase inhibitors
VD3	1,25-dihydroxyvitamin D3
8-OHD	8-hydroxydaidzein

## References

[1] Sung H., Ferlay J., Siegel R.L., Laversanne M., Soerjomataram I., Jemal A., Bray F. (2021). Global Cancer Statistics 2020: GLOBOCAN Estimates of Incidence and Mortality Worldwide for 36 Cancers in 185 Countries. CA Cancer J. Clin..

[2] Morceau F., Chateauvieux S., Orsini M., Trecul A., Dicato M., Diederich M. (2015). Natural compounds and pharmaceuticals reprogram leukemia cell differentiation pathways. Biotechnol. Adv..

[3] Chen X., Yang M., Hao W., Han J., Ma J., Wang C., Sun S., Zheng Q. (2016). Differentiation-inducing and anti-proliferative activities of isoliquiritigenin and all-trans-retinoic acid on B16F0 melanoma cells: Mechanisms profiling by RNA-seq. Gene.

[4] Pierce G.B., Verney E.L. (1961). An in vitro and in vivo study of differentiation in teratocarcinomas. Cancer.

[5] Friend C., Scher W. (1975). Stimulation by dimethyl sulfoxide of erythroid differentiation and hemoglobin synthesis in murine virus-induced leukemic cells. Ann. N. Y. Acad. Sci..

[6] Wang Z.Y., Chen Z. (2008). Acute promyelocytic leukemia: From highly fatal to highly curable. Blood.

[7] Kreider J.W., Wade D.R., Rosenthal M., Densley T. (1975). Maturation and differentiation of B16 melanoma cells induced by theophylline treatment. J. Natl. Cancer Inst..

[8] Hata K., Mukaiyama T., Tsujimura N., Sato Y., Kosaka Y., Sakamoto K., Hori K. (2006). Differentiation-inducing activity of lupane triterpenes on a mouse melanoma cell line. Cytotechnology.

[9] Alesiani D., Cicconi R., Mattei M., Montesano C., Bei R., Canini A. (2008). Cell cycle arrest and differentiation induction by 5,7-dimethoxycoumarin in melanoma cell lines. Int. J. Oncol..

[10] Dandawate P.R., Subramaniam D., Jensen R.A., Anant S. (2016). Targeting cancer stem cells and signaling pathways by phytochemicals: Novel approach for breast cancer therapy. Semin. Cancer Biol..

[11] Sudomova M., Berchova-Bimova K., Marzocco S., Liskova A., Kubatka P., Hassan S.T.S. (2021). Berberine in Human Oncogenic Herpesvirus Infections and Their Linked Cancers. Viruses.

[12] Mendez-Ferrer S., Bonnet D., Steensma D.P., Hasserjian R.P., Ghobrial I.M., Gribben J.G., Andreeff M., Krause D.S. (2020). Bone marrow niches in haematological malignancies. Nat. Rev. Cancer.

[13] Girardi T., Vicente C., Cools J., De Keersmaecker K. (2017). The genetics and molecular biology of T-ALL. Blood.

[14] Siegel R.L., Miller K.D., Jemal A. (2020). Cancer statistics, 2020. CA Cancer J. Clin..

[15] Hasanpourghadi M., Pandurangan A.K., Mustafa M.R. (2018). Modulation of oncogenic transcription factors by bioactive natural products in breast cancer. Pharmacol. Res..

[16] Tewary P., Gunatilaka A.A., Sayers T.J. (2017). Using natural products to promote caspase-8-dependent cancer cell death. Cancer Immunol. Immunother. CII.

[17] Gupta K., Chakrabarti A., Rana S., Ramdeo R., Roth B.L., Agarwal M.L., Tse W., Agarwal M.K., Wald D.N. (2011). Securinine, a myeloid differentiation agent with therapeutic potential for AML. PLoS ONE.

[18] Sharma J., Pandey A., Sharma S., Dixit A. (2022). Securinine Induces Differentiation of Human Promyelocytic Leukemic HL-60 Cells through JNK-Mediated Signaling Pathway. Nutr. Cancer.

[19] Han S., Zhang G., Li M., Chen D., Wang Y., Ye W., Ji Z. (2014). L-securinine induces apoptosis in the human promyelocytic leukemia cell line HL-60 and influences the expression of genes involved in the PI3K/AKT/mTOR signaling pathway. Oncol. Rep..

[20] Hou W., Wang Z.Y., Lin J., Chen W.M. (2020). Induction of differentiation of the acute myeloid leukemia cell line (HL-60) by a securinine dimer. Cell Death Discov..

[21] Sun J., Mu H., Yu J., Li L., Yan H., Li G., Tan H., Yang N., Yang X., Yi L. (2019). Diallyl disulfide down-regulates calreticulin and promotes C/EBPalpha expression in differentiation of human leukaemia cells. J. Cell. Mol. Med..

[22] Yi L., Shan J., Chen X., Li G., Li L., Tan H., Su Q. (2016). Involvement of calreticulin in cell proliferation, invasion and differentiation in diallyl disulfide-treated HL-60 cells. Oncol. Lett..

[23] Ling H., Ji X., Lei Y., Jia Y., Liu F., Xia H., Tan H., Zeng X., Yi L., He J. (2020). Diallyl disulfide induces downregulation and inactivation of cofilin 1 differentiation via the Rac1/ROCK1/LIMK1 pathway in leukemia cells. Int. J. Oncol..

[24] Agassi S.F.T., Yeh T.M., Chang C.D., Hsu J.L., Shih W.L. (2020). Potentiation of Differentiation and Apoptosis in a Human Promyelocytic Leukemia Cell Line by Garlic Essential Oil and Its Organosulfur Compounds. Anticancer Res..

[25] Zhang B., Chen N., Chen H., Wang Z., Zheng Q. (2012). The critical role of redox homeostasis in shikonin-induced HL-60 cell differentiation via unique modulation of the Nrf2/ARE pathway. Oxid. Med. Cell. Longev..

[26] Duan D., Zhang B., Yao J., Liu Y., Fang J. (2014). Shikonin targets cytosolic thioredoxin reductase to induce ROS-mediated apoptosis in human promyelocytic leukemia HL-60 cells. Free Radic. Biol. Med..

[27] Han W., Xie J., Fang Y., Wang Z., Pan H. (2012). Nec-1 enhances shikonin-induced apoptosis in leukemia cells by inhibition of RIP-1 and ERK1/2. Int. J. Mol. Sci..

[28] Ma H.Y., Wang C.Q., He H., Yu Z.Y., Tong Y., Liu G., Yang Y.Q., Li L., Pang L., Qi H.Y. (2020). Ethyl acetate extract of Caesalpinia sappan L. inhibited acute myeloid leukemia via ROS-mediated apoptosis and differentiation. Phytomed. Int. J. Phytother. Phytopharm..

[29] Wang Q., Salman H., Danilenko M., Studzinski G.P. (2005). Cooperation between antioxidants and 1,25-dihydroxyvitamin D3 in induction of leukemia HL60 cell differentiation through the JNK/AP-1/Egr-1 pathway. J. Cell. Physiol..

[30] Liu Z.H., Li J., Xia J., Jiang R., Zuo G.W., Li X.P., Chen Y., Xiong W., Chen D.L. (2015). Ginsenoside 20(s)-Rh2 as potent natural histone deacetylase inhibitors suppressing the growth of human leukemia cells. Chem.-Biol. Interact..

[31] Wang X., Wang Y. (2015). Ginsenoside Rh2 Mitigates Pediatric Leukemia Through Suppression of Bcl-2 in Leukemia Cells. Cell. Physiol. Biochem. Int. J. Exp. Cell. Physiol. Biochem. Pharmacol..

[32] Wang C., He H., Dou G., Li J., Zhang X., Jiang M., Li P., Huang X., Chen H., Li L. (2017). Ginsenoside 20(S)-Rh2 Induces Apoptosis and Differentiation of Acute Myeloid Leukemia Cells: Role of Orphan Nuclear Receptor Nur77. J. Agric. Food Chem..

[33] Zhuang J., Yin J., Xu C., Mu Y., Lv S. (2018). 20(S)-Ginsenoside Rh2 Induce the Apoptosis and Autophagy in U937 and K562 Cells. Nutrients.

[34] Yu Z., Li L., Wang C., He H., Liu G., Ma H., Pang L., Jiang M., Lu Q., Li P. (2020). Cantharidin Induces Apoptosis and Promotes Differentiation of AML Cells Through Nuclear Receptor Nur77-Mediated Signaling Pathway. Front. Pharmacol..

[35] Wang S.C., Chow J.M., Chien M.H., Lin C.W., Chen H.Y., Hsiao P.C., Yang S.F. (2018). Cantharidic acid induces apoptosis of human leukemic HL-60 cells via c-Jun N-terminal kinase-regulated caspase-8/-9/-3 activation pathway. Environ. Toxicol..

[36] Jiang Y.M., Meng Z.Z., Yue G.X., Chen J.X. (2012). Norcantharidin Induces HL-60 Cells Apoptosis In Vitro. Evid.-Based Complement. Altern. Med. Ecam.

[37] Chen D., Wang Q., Li Y., Sun P., Kuek V., Yuan J., Yang J., Wen L., Wang H., Xu J. (2021). Notopterol Attenuates Estrogen Deficiency-Induced Osteoporosis via Repressing RANKL Signaling and Reactive Oxygen Species. Front. Pharmacol..

[38] Wang Q., Zhou X., Yang L., Zhao Y., Chew Z., Xiao J., Liu C., Zheng X., Zheng Y., Shi Q. (2020). The Natural Compound Notopterol Binds and Targets JAK2/3 to Ameliorate Inflammation and Arthritis. Cell Rep..

[39] Jiang X., Lu H., Li J., Liu W., Wu Q., Xu Z., Qiao Q., Zhang H., Gao H., Zhao Q. (2020). A natural BACE1 and GSK3beta dual inhibitor Notopterol effectively ameliorates the cognitive deficits in APP/PS1 Alzheimer’s mice by attenuating amyloid-beta and tau pathology. Clin. Transl. Med..

[40] Huang Q., Wang L., Ran Q., Wang J., Wang C., He H., Li L., Qi H. (2019). Notopterol-induced apoptosis and differentiation in human acute myeloid leukemia HL-60 cells. Drug Des. Dev. Ther..

[41] Khan G.J., Rizwan M., Abbas M., Naveed M., Boyang Y., Naeem M.A., Khan S., Yuan S., Baig M., Sun L. (2018). Pharmacological effects and potential therapeutic targets of DT-13. Biomed. Pharmacother..

[42] Wei X., Mao T., Li S., He J., Hou X., Li H., Zhan M., Yang X., Li R., Xiao J. (2019). DT-13 inhibited the proliferation of colorectal cancer via glycolytic metabolism and AMPK/mTOR signaling pathway. Phytomed. Int. J. Phytother. Phytopharm..

[43] Du H., Liu Y., Chen X., Yu X., Hou X., Li H., Zhan M., Lin S., Lu L., Yuan S. (2018). DT-13 synergistically potentiates the sensitivity of gastric cancer cells to topotecan via cell cycle arrest in vitro and in vivo. Eur. J. Pharmacol..

[44] Wang C., He H., Liu G., Ma H., Li L., Jiang M., Lu Q., Li P., Qi H. (2020). DT-13 induced apoptosis and promoted differentiation of acute myeloid leukemia cells by activating AMPK-KLF2 pathway. Pharmacol. Res..

[45] Samadian N., Hashemi M. (2018). Effects of Apigenin and Apigenin- Loaded Nanogel on Induction of Apoptosis in Human Chronic Myeloid Leukemia Cells. Galen Med. J..

[46] Danisman Kalindemirtas F., Birman H., Candoken E., Bilgis Gazioglu S., Melikoglu G., Kuruca S. (2019). Cytotoxic Effects of Some Flavonoids and Imatinib on the K562 Chronic Myeloid Leukemia Cell Line: Data Analysis Using the Combination Index Method. Balk. Med. J..

[47] Tsolmon S., Nakazaki E., Han J., Isoda H. (2011). Apigetrin induces erythroid differentiation of human leukemia cells K562: Proteomics approach. Mol. Nutr. Food Res..

[48] Isoda H., Motojima H., Onaga S., Samet I., Villareal M.O., Han J. (2014). Analysis of the erythroid differentiation effect of flavonoid apigenin on K562 human chronic leukemia cells. Chem. Biol. Interact..

[49] Dupont C., Couillerot E., Gillet R., Caron C., Zeches-Hanrot M., Riou J.F., Trentesaux C. (2005). The benzophenanthridine alkaloid fagaronine induces erythroleukemic cell differentiation by gene activation. Planta Med..

[50] Huang B., Liu H., Huang D., Mao X., Hu X., Jiang C., Pu M., Zhang G., Zeng X. (2016). Apoptosis Induction and Imaging of Cadmium-Telluride Quantum Dots with Wogonin in Multidrug-Resistant Leukemia K562/A02 Cell. J. Nanosci. Nanotechnol..

[51] Hu C., Xu M., Qin R., Chen W., Xu X. (2015). Wogonin induces apoptosis and endoplasmic reticulum stress in HL-60 leukemia cells through inhibition of the PI3K-AKT signaling pathway. Oncol. Rep..

[52] Boozari M., Mohammadi A., Asili J., Emami S.A., Tayarani-Najaran Z. (2015). Growth inhibition and apoptosis induction by Scutellaria pinnatifida A. Ham. on HL-60 and K562 leukemic cell lines. Environ. Toxicol. Pharmacol..

[53] Yang H., Hui H., Wang Q., Li H., Zhao K., Zhou Y., Zhu Y., Wang X., You Q., Guo Q. (2020). Correction: Wogonin induces cell cycle arrest and erythroid differentiation in imatinib-resistant K562 cells and primary CML cells. Oncotarget.

[54] Zhao Y., Bilal M., Raza A., Khan M.I., Mehmood S., Hayat U., Hassan S.T.S., Iqbal H.M.N. (2021). Tyrosine kinase inhibitors and their unique therapeutic potentialities to combat cancer. Int. J. Biol. Macromol..

[55] Shi X., Chen X., Li X., Lan X., Zhao C., Liu S., Huang H., Liu N., Liao S., Song W. (2014). Gambogic acid induces apoptosis in imatinib-resistant chronic myeloid leukemia cells via inducing proteasome inhibition and caspase-dependent Bcr-Abl downregulation. Clin. Cancer Res. Off. J. Am. Assoc. Cancer Res..

[56] Chen J., Zhou M., Zhang Q., Xu J., Ouyang J. (2015). Gambogic acid induces death of K562 cells through autophagy and apoptosis mechanisms. Leuk. Lymphoma.

[57] Sun Y., Liu W.J. (2020). Research Advance on Reversing Multidrug Resistance of Chronic Myeloid Leukemia by Chinese Herbal Monomer–Review. Zhongguo Shi Yan Xue Ye Xue Za Zhi.

[58] Dai Y., Chen S.R., Chai L., Zhao J., Wang Y., Wang Y. (2019). Overview of pharmacological activities of Andrographis paniculata and its major compound andrographolide. Crit. Rev. Food Sci. Nutr..

[59] Liao H.C., Chou Y.J., Lin C.C., Liu S.H., Oswita A., Huang Y.L., Wang Y.L., Syu J.L., Sun C.M., Leu C.M. (2019). Andrographolide and its potent derivative exhibit anticancer effects against imatinib-resistant chronic myeloid leukemia cells by downregulating the Bcr-Abl oncoprotein. Biochem. Pharmacol..

[60] Liu S.H., Lin C.H., Liang F.P., Chen P.F., Kuo C.D., Alam M.M., Maiti B., Hung S.K., Chi C.W., Sun C.M. (2014). Andrographolide downregulates the v-Src and Bcr-Abl oncoproteins and induces Hsp90 cleavage in the ROS-dependent suppression of cancer malignancy. Biochem. Pharmacol..

[61] Mokenapelli S., Gutam M., Vadiyaala N., Yerrabelli J.R., Banerjee S., Roy P., Kancha R.K., Kunduru B.R., Sagurthi S.R., Chitneni P.R. (2021). Synthesis and cytotoxicity of novel 14alpha-O-(1,4-disubstituted-1,2,3-triazolyl) ester derivatives of andrographolide. Nat. Prod. Res..

[62] Wu P.S., Yen J.H., Wang C.Y., Chen P.Y., Hung J.H., Wu M.J. (2020). 8-Hydroxydaidzein, an Isoflavone from Fermented Soybean, Induces Autophagy, Apoptosis, Differentiation, and Degradation of Oncoprotein BCR-ABL in K562 Cells. Biomedicines.

[63] Wu P.S., Wang C.Y., Chen P.S., Hung J.H., Yen J.H., Wu M.J. (2021). 8-Hydroxydaidzein Downregulates JAK/STAT, MMP, Oxidative Phosphorylation, and PI3K/AKT Pathways in K562 Cells. Biomedicines.

[64] Huang H.L., Lin T.W., Huang Y.L., Huang R.L. (2016). Induction of apoptosis and differentiation by atractylenolide-1 isolated from Atractylodes macrocephala in human leukemia cells. Bioorg. Med. Chem. Lett..

[65] Piechotta V., Jakob T., Langer P., Monsef I., Scheid C., Estcourt L.J., Ocheni S., Theurich S., Kuhr K., Scheckel B. (2019). Multiple drug combinations of bortezomib, lenalidomide, and thalidomide for first-line treatment in adults with transplant-ineligible multiple myeloma: A network meta-analysis. Cochrane Database Syst. Rev..

[66] Carpenter R.S., Goodrich L.R., Frisbie D.D., Kisiday J.D., Carbone B., McIlwraith C.W., Centeno C.J., Hidaka C. (2010). Osteoblastic differentiation of human and equine adult bone marrow-derived mesenchymal stem cells when BMP-2 or BMP-7 homodimer genetic modification is compared to BMP-2/7 heterodimer genetic modification in the presence and absence of dexamethasone. J. Orthop. Res. Off. Publ. Orthop. Res. Soc..

[67] Ema H., Morita Y., Suda T. (2014). Heterogeneity and hierarchy of hematopoietic stem cells. Exp. Hematol..

[68] Ying X., Sun L., Chen X., Xu H., Guo X., Chen H., Hong J., Cheng S., Peng L. (2013). Silibinin promotes osteoblast differentiation of human bone marrow stromal cells via bone morphogenetic protein signaling. Eur. J. Pharmacol..

[69] Kihara T., Ichikawa S., Yonezawa T., Lee J.W., Akihisa T., Woo J.T., Michi Y., Amagasa T., Yamaguchi A. (2011). Acerogenin A, a natural compound isolated from Acer nikoense Maxim, stimulates osteoblast differentiation through bone morphogenetic protein action. Biochem. Biophys. Res. Commun..

[70] Yonezawa T., Lee J.W., Akazawa H., Inagaki M., Cha B.Y., Nagai K., Yagasaki K., Akihisa T., Woo J.T. (2011). Osteogenic activity of diphenyl ether-type cyclic diarylheptanoids derived from Acer nikoense. Bioorg. Med. Chem. Lett..

[71] Kim J.M., Lee S.U., Kim Y.S., Min Y.K., Kim S.H. (2008). Baicalein stimulates osteoblast differentiation via coordinating activation of MAP kinases and transcription factors. J. Cell. Biochem..

[72] Yu C.C., Li Y., Cheng Z.J., Wang X., Mao W., Zhang Y.W. (2022). Active Components of Traditional Chinese Medicinal Material for Multiple Myeloma: Current Evidence and Future Directions. Front. Pharmacol..

[73] Banik K., Khatoon E., Harsha C., Rana V., Parama D., Thakur K.K., Bishayee A., Kunnumakkara A.B. (2022). Wogonin and its analogs for the prevention and treatment of cancer: A systematic review. Phytother. Res. PTR.

[74] Li S.F., Tang J.J., Chen J., Zhang P., Wang T., Chen T.Y., Yan B., Huang B., Wang L., Huang M.J. (2015). Regulation of bone formation by baicalein via the mTORC1 pathway. Drug Des. Dev. Ther..

[75] Tian X., Jiang H., Chen Y., Ao X., Chen C., Zhang W., He F., Liao X., Jiang X., Li T. (2018). Baicalein Accelerates Tendon-Bone Healing via Activation of Wnt/beta-Catenin Signaling Pathway in Rats. BioMed Res. Int..

[76] Boissy P., Andersen T.L., Abdallah B.M., Kassem M., Plesner T., Delaisse J.M. (2005). Resveratrol inhibits myeloma cell growth, prevents osteoclast formation, and promotes osteoblast differentiation. Cancer Res..

[77] Kupisiewicz K., Boissy P., Abdallah B.M., Hansen F.D., Erben R.G., Savouret J.F., Soe K., Andersen T.L., Plesner T., Delaisse J.M. (2010). Potential of resveratrol analogues as antagonists of osteoclasts and promoters of osteoblasts. Calcif. Tissue Int..

[78] Ma R., Yu D., Peng Y., Yi H., Wang Y., Cheng T., Shi B., Yang G., Lai W., Wu X. (2021). Resveratrol induces AMPK and mTOR signaling inhibition-mediated autophagy and apoptosis in multiple myeloma cells. Acta Biochim. Biophys. Sin..

[79] Li Q., Yue Y., Chen L., Xu C., Wang Y., Du L., Xue X., Liu Q., Wang Y., Fan F. (2018). Resveratrol Sensitizes Carfilzomib-Induced Apoptosis via Promoting Oxidative Stress in Multiple Myeloma Cells. Front. Pharmacol..

[80] Yang X.J., Xi Y.M., Li Z.J. (2019). Icaritin: A Novel Natural Candidate for Hematological Malignancies Therapy. BioMed Res. Int..

[81] Ma X.N., Ma C.X., Shi W.G., Zhou J., Ma H.P., Gao Y.H., Xian C.J., Chen K.M. (2017). Primary cilium is required for the stimulating effect of icaritin on osteogenic differentiation and mineralization of osteoblasts in vitro. J. Endocrinol. Investig..

[82] Wu T., Shu T., Kang L., Wu J., Xing J., Lu Z., Chen S., Lv J. (2017). Icaritin, a novel plant-derived osteoinductive agent, enhances the osteogenic differentiation of human bone marrow- and human adipose tissue-derived mesenchymal stem cells. Int. J. Mol. Med..

[83] Wu Z., Ou L., Wang C., Yang L., Wang P., Liu H., Xiong Y., Sun K., Zhang R., Zhu X. (2017). Icaritin induces MC3T3-E1 subclone14 cell differentiation through estrogen receptor-mediated ERK1/2 and p38 signaling activation. Biomed. Pharmacother..

[84] Zhu J., Li Z., Zhang G., Meng K., Kuang W., Li J., Zhou X., Li R., Peng H., Dai C. (2011). Icaritin shows potent anti-leukemia activity on chronic myeloid leukemia in vitro and in vivo by regulating MAPK/ERK/JNK and JAK2/STAT3 /AKT signalings. PLoS ONE.

[85] Tang N., Song W.X., Luo J., Haydon R.C., He T.C. (2008). Osteosarcoma development and stem cell differentiation. Clin. Orthop. Relat. Res..

[86] Longhi A., Errani C., De Paolis M., Mercuri M., Bacci G. (2006). Primary bone osteosarcoma in the pediatric age: State of the art. Cancer Treat. Rev..

[87] Ying M., Liu G., Shimada H., Ding W., May W.A., He Q., Adams G.B., Wu L. (2013). Human osteosarcoma CD49f(-)CD133(+) cells: Impaired in osteogenic fate while gain of tumorigenicity. Oncogene.

[88] Ferguson W.S., Goorin A.M. (2001). Current treatment of osteosarcoma. Cancer Investig..

[89] Liskova A., Samec M., Koklesova L., Brockmueller A., Zhai K., Abdellatif B., Siddiqui M., Biringer K., Kudela E., Pec M. (2021). Flavonoids as an effective sensitizer for anti-cancer therapy: Insights into multi-faceted mechanisms and applicability towards individualized patient profiles. EPMA J..

[90] Pang X.G., Cong Y., Bao N.R., Li Y.G., Zhao J.N. (2018). Quercetin Stimulates Bone Marrow Mesenchymal Stem Cell Differentiation through an Estrogen Receptor-Mediated Pathway. BioMed Res. Int..

[91] Maleki Dana P., Sadoughi F., Asemi Z., Yousefi B. (2021). Anti-cancer properties of quercetin in osteosarcoma. Cancer Cell Int..

[92] Li S., Pei Y., Wang W., Liu F., Zheng K., Zhang X. (2019). Quercetin suppresses the proliferation and metastasis of metastatic osteosarcoma cells by inhibiting parathyroid hormone receptor 1. Biomed. Pharmacother..

[93] Casado-Diaz A., Anter J., Dorado G., Quesada-Gomez J.M. (2016). Effects of quercetin, a natural phenolic compound, in the differentiation of human mesenchymal stem cells (MSC) into adipocytes and osteoblasts. J. Nutr. Biochem..

[94] Zhang D., Fong C., Jia Z., Cui L., Yao X., Yang M. (2016). Icariin Stimulates Differentiation and Suppresses Adipocytic Transdifferentiation of Primary Osteoblasts Through Estrogen Receptor-Mediated Pathway. Calcif. Tissue Int..

[95] Ren Y., Zhu F., Liu Z. (2018). Inhibitory effect of icariin on osteosarcoma cell proliferation via the Wnt/beta-catenin signaling pathway. Oncol. Lett..

[96] Wang Z.D., Wang R.Z., Xia Y.Z., Kong L.Y., Yang L. (2018). Reversal of multidrug resistance by icaritin in doxorubicin-resistant human osteosarcoma cells. Chin. J. Nat. Med..

[97] Sun L.J., Li C., Wen X.H., Guo L., Guo Z.F., Liao L.Q., Guo Y. (2021). Icariin Stimulates hFOB 1.19 Osteoblast Proliferation and Differentiation via OPG/RANKL Mediated by the Estrogen Receptor. Curr. Pharm. Biotechnol..

[98] Xu Y., Li L., Tang Y., Yang J., Jin Y., Ma C. (2019). Icariin promotes osteogenic differentiation by suppressing Notch signaling. Eur. J. Pharmacol..

[99] Dai J., Li Y., Zhou H., Chen J., Chen M., Xiao Z. (2013). Genistein promotion of osteogenic differentiation through BMP2/SMAD5/RUNX2 signaling. Int. J. Biol. Sci..

[100] Engel N., Adamus A., Schauer N., Kuhn J., Nebe B., Seitz G., Kraft K. (2017). Synergistic Action of Genistein and Calcitriol in Immature Osteosarcoma MG-63 Cells by SGPL1 Up-Regulation. PLoS ONE.

[101] Song M., Tian X., Lu M., Zhang X., Ma K., Lv Z., Wang Z., Hu Y., Xun C., Zhang Z. (2015). Genistein exerts growth inhibition on human osteosarcoma MG-63 cells via PPARgamma pathway. Int. J. Oncol..

[102] Sarkar N., Bose S. (2020). Controlled release of soy isoflavones from multifunctional 3D printed bone tissue engineering scaffolds. Acta Biomater..

[103] Guo A.J., Choi R.C., Zheng K.Y., Chen V.P., Dong T.T., Wang Z.T., Vollmer G., Lau D.T., Tsim K.W. (2012). Kaempferol as a flavonoid induces osteoblastic differentiation via estrogen receptor signaling. Chin. Med..

[104] Huang W.W., Chiu Y.J., Fan M.J., Lu H.F., Yeh H.F., Li K.H., Chen P.Y., Chung J.G., Yang J.S. (2010). Kaempferol induced apoptosis via endoplasmic reticulum stress and mitochondria-dependent pathway in human osteosarcoma U-2 OS cells. Mol. Nutr. Food Res..

[105] Chen J., Teng J., Ma L., Tong H., Ren B., Wang L., Li W. (2017). Flavonoids Isolated From the Flowers of Limonium bicolor and their In vitro Antitumor Evaluation. Pharmacogn. Mag..

[106] Lee C.H., Huang Y.L., Liao J.F., Chiou W.F. (2012). Ugonin K-stimulated osteogenesis involves estrogen receptor-dependent activation of non-classical Src signaling pathway and classical pathway. Eur. J. Pharmacol..

[107] Lee C.H., Huang Y.L., Liao J.F., Chiou W.F. (2011). Ugonin K promotes osteoblastic differentiation and mineralization by activation of p38 MAPK- and ERK-mediated expression of Runx2 and osterix. Eur. J. Pharmacol..

[108] Liu C., Ma M., Zhang J., Gui S., Zhang X., Xue S. (2017). Galangin inhibits human osteosarcoma cells growth by inducing transforming growth factor-beta1-dependent osteogenic differentiation. Biomed. Pharmacother..

[109] Yang Z., Li X., Han W., Lu X., Jin S., Yang W., Li J., He W., Qian Y. (2017). Galangin suppresses human osteosarcoma cells: An exploration of its underlying mechanism. Oncol. Rep..

[110] Huh J.E., Jung I.T., Choi J., Baek Y.H., Lee J.D., Park D.S., Choi D.Y. (2013). The natural flavonoid galangin inhibits osteoclastic bone destruction and osteoclastogenesis by suppressing NF-kappaB in collagen-induced arthritis and bone marrow-derived macrophages. Eur. J. Pharmacol..

[111] Zhang N., Ying M.D., Wu Y.P., Zhou Z.H., Ye Z.M., Li H., Lin D.S. (2014). Hyperoside, a flavonoid compound, inhibits proliferation and stimulates osteogenic differentiation of human osteosarcoma cells. PLoS ONE.

[112] Qi X.C., Li B., Wu W.L., Liu H.C., Jiang Y.P. (2020). Protective effect of hyperoside against hydrogen peroxide-induced dysfunction and oxidative stress in osteoblastic MC3T3-E1 cells. Artif. Cells Nanomed. Biotechnol..

[113] Zhang Q., Zhang X.F. (2019). Hyperoside decreases the apoptosis and autophagy rates of osteoblast MC3T3E1 cells by regulating TNFlike weak inducer of apoptosis and the p38mitogen activated protein kinase pathway. Mol. Med. Rep..

[114] Geng S., Sun B., Lu R., Wang J. (2014). Coleusin factor, a novel anticancer diterpenoid, inhibits osteosarcoma growth by inducing bone morphogenetic protein-2-dependent differentiation. Mol. Cancer Ther..

[115] Kommagani R., Whitlatch A., Leonard M.K., Kadakia M.P. (2010). p73 is essential for vitamin D-mediated osteoblastic differentiation. Cell Death Differ..

[116] Wang D., Song J., Ma H. (2018). An in vitro Experimental Insight into the Osteoblast Responses to Vitamin D3 and Its Metabolites. Pharmacology.

[117] Nagpal S., Na S., Rathnachalam R. (2005). Noncalcemic actions of vitamin D receptor ligands. Endocr. Rev..

[118] Tahbazlahafi B., Paknejad M., Khaghani S., Sadegh-Nejadi S., Khalili E. (2021). Vitamin D Represses the Aggressive Potential of Osteosarcoma. Endocr. Metab. Immune Disord. Drug Targets.

[119] Samuel S., Sitrin M.D. (2008). Vitamin D’s role in cell proliferation and differentiation. Nutr. Rev..

[120] Gombart A.F., Luong Q.T., Koeffler H.P. (2006). Vitamin D compounds: Activity against microbes and cancer. Anticancer Res..

[121] Cordella M., Tabolacci C., Senatore C., Rossi S., Mueller S., Lintas C., Eramo A., D’Arcangelo D., Valitutti S., Facchiano A. (2019). Theophylline induces differentiation and modulates cytoskeleton dynamics and cytokines secretion in human melanoma-initiating cells. Life Sci..

[122] Huang H.C., Yen H., Lu J.Y., Chang T.M., Hii C.H. (2020). Theophylline enhances melanogenesis in B16F10 murine melanoma cells through the activation of the MEK 1/2, and Wnt/beta-catenin signaling pathways. Food Chem. Toxicol. Int. J. Publ. Br. Ind. Biol. Res. Assoc..

[123] Wang W., Zhang Y., Nakashima S., Nakamura S., Wang T., Yoshikawa M., Matsuda H. (2019). Inhibition of melanin production by anthracenone dimer glycosides isolated from Cassia auriculata seeds. J. Nat. Med..

[124] Xiang S., Zeng H., Xia F., Ji Q., Xue J., Ren R., Que F., Zhou B. (2021). The dietary flavonoid isoliquiritigenin induced apoptosis and suppressed metastasis in melanoma cells: An in vitro and in vivo study. Life Sci..

[125] Xiang S., Chen H., Luo X., An B., Wu W., Cao S., Ruan S., Wang Z., Weng L., Zhu H. (2018). Isoliquiritigenin suppresses human melanoma growth by targeting miR-301b/LRIG1 signaling. J. Exp. Clin. Cancer Res. CR.

[126] Chen X.Y., Ren H.H., Wang D., Chen Y., Qu C.J., Pan Z.H., Liu X.N., Hao W.J., Xu W.J., Wang K.J. (2019). Isoliquiritigenin Induces Mitochondrial Dysfunction and Apoptosis by Inhibiting mitoNEET in a Reactive Oxygen Species-Dependent Manner in A375 Human Melanoma Cells. Oxid. Med. Cell. Longev..

[127] Qiang D., Ci C., Liu W., Wang J., He C., Ji B., Shao X. (2021). Inhibitory effect of kaempferol on mouse melanoma cell line B16 in vivo and in vitro. Postepy Dermatol. I Alergol..

[128] Yang J., Xiao P., Sun J., Guo L. (2018). Anticancer effects of kaempferol in A375 human malignant melanoma cells are mediated via induction of apoptosis, cell cycle arrest, inhibition of cell migration and downregulation of m-TOR/PI3K/AKT pathway. J. BUON Off. J. Balk. Union Oncol..

[129] Bouhlel Chatti I., Ben Toumia I., Krichen Y., Maatouk M., Chekir Ghedira L., Krifa M. (2021). Assessment of Rhamnus alaternus Leaves Extract: Phytochemical Characterization and Antimelanoma Activity. J. Med. Food.

[130] Sabitov A., Gawel-Beben K., Sakipova Z., Strzepek-Gomolka M., Hoian U., Satbayeva E., Glowniak K., Ludwiczuk A. (2021). Rosa platyacantha Schrenk from Kazakhstan-Natural Source of Bioactive Compounds with Cosmetic Significance. Molecules.

[131] Heriniaina R.M., Dong J., Kalavagunta P.K., Wu H.L., Yan D.S., Shang J. (2018). Effects of six compounds with different chemical structures on melanogenesis. Chin. J. Nat. Med..

[132] Cui S., Wang J., Wu Q., Qian J., Yang C., Bo P. (2017). Genistein inhibits the growth and regulates the migration and invasion abilities of melanoma cells via the FAK/paxillin and MAPK pathways. Oncotarget.

[133] Venza I., Visalli M., Oteri R., Beninati C., Teti D., Venza M. (2018). Genistein reduces proliferation of EP3-expressing melanoma cells through inhibition of PGE2-induced IL-8 expression. Int. Immunopharmacol..

[134] Chiang C.M., Chang Y.J., Wu J.Y., Chang T.S. (2017). Production and Anti-Melanoma Activity of Methoxyisoflavones from the Biotransformation of Genistein by Two Recombinant Escherichia coli Strains. Molecules.

[135] Wang X., Zhao Y., Yu M., Xu Y. (2020). PTEN/Akt Signaling-Mediated Activation of the Mitochondrial Pathway Contributes to the 3,3′-Diindolylmethane-Mediated Antitumor Effect in Malignant Melanoma Cells. J. Med. Food.

[136] Maciejewska D., Rasztawicka M., Wolska I., Anuszewska E., Gruber B. (2009). Novel 3,3′-diindolylmethane derivatives: Synthesis and cytotoxicity, structural characterization in solid state. Eur. J. Med. Chem..

[137] Heo J.R., Lee G.A., Kim G.S., Hwang K.A., Choi K.C. (2018). Phytochemical-induced reactive oxygen species and endoplasmic reticulum stress-mediated apoptosis and differentiation in malignant melanoma cells. Phytomed. Int. J. Phytother. Phytopharm..

[138] Colon N.C., Chung D.H. (2011). Neuroblastoma. Adv. Pediatr..

[139] Abdullah A., Talwar P., d’Hellencourt C.L., Ravanan P. (2019). IRE1alpha is critical for Kaempferol-induced neuroblastoma differentiation. FEBS J..

[140] Pham H.N.T., Sakoff J.A., Vuong Q.V., Bowyer M.C., Scarlett C.J. (2018). Comparative cytotoxic activity between kaempferol and gallic acid against various cancer cell lines. Data Brief.

[141] Lee W.J., Chen L.C., Lin J.H., Cheng T.C., Kuo C.C., Wu C.H., Chang H.W., Tu S.H., Ho Y.S. (2019). Melatonin promotes neuroblastoma cell differentiation by activating hyaluronan synthase 3-induced mitophagy. Cancer Med..

[142] Pourhanifeh M.H., Kamali M., Mehrzadi S., Hosseinzadeh A. (2021). Melatonin and neuroblastoma: A novel therapeutic approach. Mol. Biol. Rep..

[143] Shukla M., Chinchalongporn V., Govitrapong P. (2020). Melatonin Prevents Neddylation Dysfunction in Abeta42-Exposed SH-SY5Y Neuroblastoma Cells by Regulating the Amyloid Precursor Protein- Binding Protein 1 Pathway. Curr. Alzheimer Res..

[144] Song W.J., Yun J.H., Jeong M.S., Kim K.N., Shin T., Kim H.C., Wie M.B. (2021). Inhibitors of Lipoxygenase and Cyclooxygenase-2 Attenuate Trimethyltin-Induced Neurotoxicity through Regulating Oxidative Stress and Pro-Inflammatory Cytokines in Human Neuroblastoma SH-SY5Y Cells. Brain Sci..

[145] Nopparat C., Chaopae W., Boontem P., Sopha P., Wongchitrat P., Govitrapong P. (2021). Melatonin Attenuates High Glucose-Induced Changes in Beta Amyloid Precursor Protein Processing in Human Neuroblastoma Cells. Neurochem. Res..

[146] Mishra R., Kaur G. (2015). Tinospora cordifolia Induces Differentiation and Senescence Pathways in Neuroblastoma Cells. Mol. Neurobiol..

[147] Sharma A., Saggu S.K., Mishra R., Kaur G. (2019). Anti-brain cancer activity of chloroform and hexane extracts of Tinospora cordifolia Miers: An in vitro perspective. Ann. Neurosci..

[148] Huang S., Wang L.L., Xue N.N., Li C., Guo H.H., Ren T.K., Zhan Y., Li W.B., Zhang J., Chen X.G. (2019). Chlorogenic acid effectively treats cancers through induction of cancer cell differentiation. Theranostics.

[149] Hall S., Anoopkumar-Dukie S., Grant G.D., Desbrow B., Lai R., Arora D., Hong Y. (2017). Modulation of chemotherapy-induced cytotoxicity in SH-SY5Y neuroblastoma cells by caffeine and chlorogenic acid. Toxicol. Mech. Methods.

[150] Prommaban A., Utama-Ang N., Chaikitwattana A., Uthaipibull C., Porter J.B., Srichairatanakool S. (2020). Phytosterol, Lipid and Phenolic Composition, and Biological Activities of Guava Seed Oil. Molecules.

[151] Zhao Z., Bo Z., Gong W., Guo Y. (2020). Inhibitor of Differentiation 1 (Id1) in Cancer and Cancer Therapy. Int. J. Med. Sci..

[152] Sato A., Okada M., Shibuya K., Watanabe E., Seino S., Suzuki K., Narita Y., Shibui S., Kayama T., Kitanaka C. (2013). Resveratrol promotes proteasome-dependent degradation of Nanog via p53 activation and induces differentiation of glioma stem cells. Stem Cell Res..

[153] Li H., Liu Y., Jiao Y., Guo A., Xu X., Qu X., Wang S., Zhao J., Li Y., Cao Y. (2016). Resveratrol sensitizes glioblastoma-initiating cells to temozolomide by inducing cell apoptosis and promoting differentiation. Oncol. Rep..

[154] Castino R., Pucer A., Veneroni R., Morani F., Peracchio C., Lah T.T., Isidoro C. (2011). Resveratrol reduces the invasive growth and promotes the acquisition of a long-lasting differentiated phenotype in human glioblastoma cells. J. Agric. Food Chem..

[155] Contreras-Ochoa C.O., Lopez-Arellano M.E., Roblero-Bartolon G., Diaz-Chavez J., Moreno-Banda G.L., Reyna-Figueroa J., Munguia-Moreno J.A., Madrid-Marina V., Lagunas-Martinez A. (2020). Molecular mechanisms of cell death induced in glioblastoma by experimental and antineoplastic drugs: New and old drugs induce apoptosis in glioblastoma. Hum. Exp. Toxicol..

[156] Tovilovic-Kovacevic G., Krstic-Milosevic D., Vinterhalter B., Toljic M., Perovic V., Trajkovic V., Harhaji-Trajkovic L., Zogovic N. (2018). Xanthone-rich extract from Gentiana dinarica transformed roots and its active component norswertianin induce autophagy and ROS-dependent differentiation of human glioblastoma cell line. Phytomed. Int. J. Phytother. Phytopharm..

[157] Krstic-Milosevic D., Banjac N., Jankovic T., Eler K., Vinterhalter B. (2020). Gentiana clusii Perr.&Song.: Enhanced production of secondary metabolites by in vitro propagation. Plant Physiol. Biochem. PPB.

[158] Benameur T., Giacomucci G., Panaro M.A., Ruggiero M., Trotta T., Monda V., Pizzolorusso I., Lofrumento D.D., Porro C., Messina G. (2021). New Promising Therapeutic Avenues of Curcumin in Brain Diseases. Molecules.

[159] Ryskalin L., Biagioni F., Busceti C.L., Lazzeri G., Frati A., Fornai F. (2020). The Multi-Faceted Effect of Curcumin in Glioblastoma from Rescuing Cell Clearance to Autophagy-Independent Effects. Molecules.

[160] Zhuang W., Long L., Zheng B., Ji W., Yang N., Zhang Q., Liang Z. (2012). Curcumin promotes differentiation of glioma-initiating cells by inducing autophagy. Cancer Sci..

[161] Kim T.J., Kwon H.S., Kang M., Leem H.H., Lee K.H., Kim D.Y. (2018). The Antitumor Natural Compound Falcarindiol Disrupts Neural Stem Cell Homeostasis by Suppressing Notch Pathway. Int. J. Mol. Sci..

[162] Reiss M., Gamba-Vitalo C., Sartorelli A.C. (1986). Induction of tumor cell differentiation as a therapeutic approach: Preclinical models for hematopoietic and solid neoplasms. Cancer Treat. Rep..

[163] Newman D.J., Cragg G.M. (2012). Natural products as sources of new drugs over the 30 years from 1981 to 2010. J. Nat. Prod..

[164] Li C.Q., Lei H.M., Hu Q.Y., Li G.H., Zhao P.J. (2021). Recent Advances in the Synthetic Biology of Natural Drugs. Front. Bioeng. Biotechnol..

[165] Robles-Fernandez I., Rodriguez-Serrano F., Alvarez P.J., Ortiz R., Rama A.R., Prados J., Melguizo C., Alvarez-Manzaneda E., Aranega A. (2013). Antitumor properties of natural compounds and related molecules. Recent Pat. Anti-Cancer Drug Discov..

